# Validating a Novel 2D to 3D Knee Reconstruction Method on Preoperative Total Knee Arthroplasty Patient Anatomies

**DOI:** 10.3390/jcm13051255

**Published:** 2024-02-22

**Authors:** Shai Factor, Ron Gurel, Dor Dan, Guy Benkovich, Amit Sagi, Artsiom Abialevich, Vadim Benkovich

**Affiliations:** 1 Division of Orthopedic Surgery, Tel Aviv Medical Center, Faculty of Medicine, Tel Aviv University, Tel Aviv 6423906, Israel; 2 Orthopedic Department, Meir Medical Center, Faculty of Medicine, Tel Aviv University, Tel Aviv 4428164, Israel; 3 Orthopedic Department, Sheba Medical Center, Faculty of Medicine, Tel Aviv University, Tel Aviv 5262000, Israel; 4 Orthopedic Department, Barzilai Medical Center, Ashkelon 78278, Israel; 5 Faculty of Health Sciences, Ben-Gurion University of the Negev, Beer Sheva 8499000, Israel; 6 South West London Elective Orthopaedic Centre, Epsom KT18 7EG, UK; 7 Department of Orthopedic Surgery, Soroka Medical Center, Beer Sheva 84101, Israel; 8 Israeli Joint Health Center, Tel Aviv 69710, Israel

**Keywords:** knee reconstruction, accuracy assessment, 2D-to-3D, total knee arthroplasty (TKA), registration, artificial intelligence, 3D reconstruction, RSIP XPlan.ai

## Abstract

Background: As advanced technology continues to evolve, incorporating robotics into surgical procedures has become imperative for precision and accuracy in preoperative planning. Nevertheless, the integration of three-dimensional (3D) imaging into these processes presents both financial considerations and potential patient safety concerns. This study aims to assess the accuracy of a novel 2D-to-3D knee reconstruction solution, RSIP XPlan.ai™ (RSIP Vision, Jerusalem, Israel), on preoperative total knee arthroplasty (TKA) patient anatomies. Methods: Accuracy was calculated by measuring the Root Mean Square Error (RMSE) between X-ray-based 3D bone models generated by the algorithm and corresponding CT bone segmentations (distances of each mesh vertex to the closest vertex in the second mesh). The RMSE was computed globally for each bone, locally for eight clinically relevant bony landmark regions, and along simulated bone cut contours. In addition, the accuracies of three anatomical axes were assessed by comparing angular deviations to inter- and intra-observer baseline values. Results: The global RMSE was 0.93 ± 0.25 mm for the femur and 0.88 ± 0.14 mm for the tibia. Local RMSE values for bony landmark regions were 0.51 ± 0.33 mm for the five femoral landmarks and 0.47 ± 0.17 mm for the three tibial landmarks. The RMSE along simulated cut contours was 0.75 ± 0.35 mm for the distal femur cut and 0.63 ± 0.27 mm for the proximal tibial cut. Anatomical axial average angular deviations were 1.89° for the trans epicondylar axis (with an inter- and intra-observer baseline of 1.43°), 1.78° for the posterior condylar axis (with a baseline of 1.71°), and 2.82° (with a baseline of 2.56°) for the medial–lateral transverse axis. Conclusions: The study findings demonstrate promising results regarding the accuracy of XPlan.ai™ in reconstructing 3D bone models from plain-film X-rays. The observed accuracy on real-world TKA patient anatomies in anatomically relevant regions, including bony landmarks, cut contours, and axes, suggests the potential utility of this method in various clinical scenarios. Further validation studies on larger cohorts are warranted to fully assess the reliability and generalizability of our results. Nonetheless, our findings lay the groundwork for potential advancements in future robotic arthroplasty technologies, with XPlan.ai™ offering a promising alternative to conventional CT scans in certain clinical contexts.

## 1. Introduction

In modern orthopedics, precise preoperative planning is essential for total knee arthroplasty (TKA), often relying on three-dimensional (3D) surgical planning through computed tomography (CT) scans and robotic systems like Stryker’s Mako^TM^ [1,2]. While CT-based planning is highly accurate, it presents challenges such as financial burdens, longer turnaround times, increased patient radiation exposure, and logistical complexities. Additionally, evolving healthcare reimbursement models in the United States may limit insurance coverage for CT scans, reducing patient access to these advanced technologies [3].

Precise preoperative planning is critical for TKA to avoid implant sizing and alignment complications. Radiographs play a pivotal role in this process, with 3D CT-based approaches offering practical advantages. According to multiple studies, 3D CT planning of the knees offers advantages in accuracy over 2D knee radiographs and is required for certain robotic workflows. However, it comes with higher costs. Additionally, a major shortcoming of CT scans is that they are taken in non-weight-bearing positions, which fail to capture pathologies related to joint space narrowing while bearing weight.

Personalized surgical instruments (PSIs) have also shown success in total knee replacement in comparison to conventional techniques. A PSI utilizes disposable cutting blocks customized to match a patient’s anatomy, eliminating the need for invasive pins and instrumentation of the intramedullary canals. However, a PSI’s success also depends on accurate digital bone surface data obtained from preoperative CT or MRI scans [4,5,6,7,8].

Efforts have been made to explore X-ray-based two-dimensional-to-three-dimensional (2D–3D) reconstruction techniques for knee anatomy. Still, adoption has been slow due to concerns about accuracy in real-world pathological TKA cases. Most validation studies have been limited to healthy knee cadaver specimens, raising doubts about their applicability in clinical practice [9].

Recent advancements in artificial intelligence (AI) have paved the way for innovative algorithms that can construct 3D bone reconstructions from 2D biplanar radiographs, offering a cost-effective alternative to traditional 3D imaging. These algorithms are based on statistical shape modeling (SSM) and require specialized calibration procedures and image acquisition protocols [10,11,12]. To address these challenges, researchers have explored alternative approaches using SSM and multiple calibrated radiographic images to reconstruct patient-specific 3D surface models. SSM methods provide advantages by transforming morphological characteristics into a space governed by fundamental modes of variation. The quality of the results depends on the extent of morphological variability within the training dataset in addition to the quantity and orientation of the Digitally Reconstructed Radiographs (DRRs) [13,14]. RSIP XPlan.ai™ [15] represents a groundbreaking solution driven by AI principles. This innovation combines machine learning models, trained on a diverse dataset of over 1000 pathological knee samples, with a robust 3D calibration technique for X-ray acquisition geometry. This study’s aim was to assess the accuracy of this novel 2D-to-3D knee reconstruction solution using clinically relevant data from a TKA patient cohort, comparing X-ray-based 3D reconstructions with corresponding patient CT scans.

## 2. Materials and Methods

**Figure 1:**
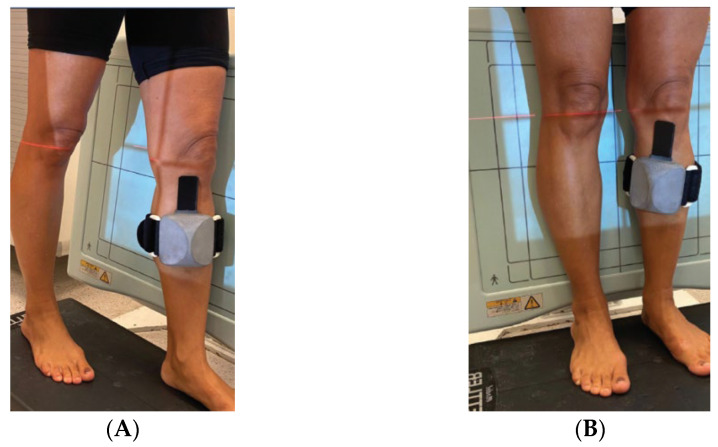
RSIP XPlan.ai™ calibration jig positioning. (**A**) Patient’s pose for lateral X-ray. (**B**) Patient’s pose for anteroposterior X-ray.

The study took place at a major medical center, was approved by the institution’s IRB, and followed the applicable laws and regulations. The inclusion criteria for the study were adults (age > 18 years old) with diagnosed osteoarthritis referred to CT scans by their orthopedic surgeon. The average within-slice resolution of the CT scans was 0.49 mm (median: 0.37 mm; STD: 0.17 mm; max: 0.71 mm), and the average slice thickness was 0.61 mm (median: 0.6 mm; STD: 0.24 mm; max: 1 mm). All patients underwent standard anteroposterior (AP) and lateral knee X-rays while wearing the RSIP XPlan.ai™ calibration jig, which was strapped to the patient’s shin by the X-ray technician (Figure 1). The pixel size of all X-ray images was 0.14 mm.

X-ray-based 3D reconstructions were generated by running the RSIP XPlan.ai™ algorithm and its associated image processing workflow. The algorithm generates 3D models from X-rays using a proprietary neural network. 

The bone model generated by the neural network is represented as a 3D volumetric mask. This mask is then converted into an STL mesh using Marching Cubes. All the CT scans were manually segmented, yielding a 3D volumetric mask for each bone. These masks are then converted into STL meshes using Marching Cubes. The segmentation was performed under the guidance of a certified radiologist or orthopedic surgeon, using ITK SNAP software (version 4.0.2), generating the ground-truth CT-based bone models. All voxels with the appearance of bone, including spongy bone, cortical bone, and osteophytes, were included in the bone segmentations; femurs and tibias were each segmented separately. 

**Figure 2:**
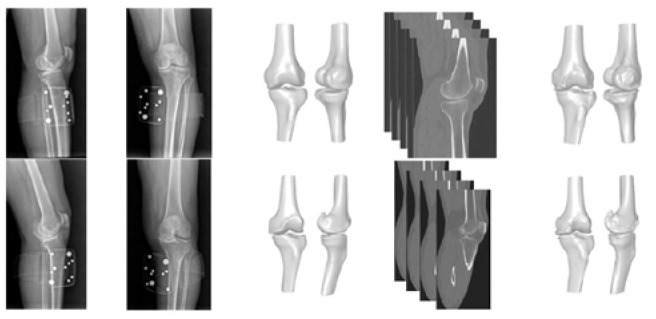
Comparison of X-ray-based 3D reconstructions and CT-based ground-truth segmentations for two patients. Each row shows data from one patient. First column: lateral X-ray. Second column: anteroposterior X-ray. Third column: X-ray-based 3D reconstruction generated by the RSIP XPlan.ai™ pipeline. Fourth column: representative view of a patient’s CT scan. Fifth column: ground-truth CT segmentation model.

Examples of the X-ray-based 3D reconstructions and their corresponding CT-based ground-truth models are shown in Figure 2. 

The Root Mean Square Error (RMSE) distance between the points of the X-ray-based 3D models and the CT segmentations was computed by registering the STL meshes using the Iterative Closest Points algorithm. Subsequently, the RMSE was calculated by taking the square root of the mean of the square distances of each mesh vertex to the closest vertex in the second mesh. This computation was performed using the Python implementation of Open3D (version 0.17.0), using the following formula:

$$RMSE=\sqrt{\sum_{i=1}^{N}\frac{{\left({\hat{y}}_{i}-y_{i}\right)}^{2}}{N}}\;$$

${\hat{y}}_{i}$ = predicted surface point;
$y_{i}$ = actual surface point;
N = # of points (For X-ray-based models.)

The average number of points (N) was 34,298 (STD: 4669) for the femur, and 29,852 (STD: 4463) for the tibia.

Eight bony landmarks (five on the femur and three on the tibia) were analyzed to assess reconstruction accuracy in clinically relevant regions, as illustrated in Table 1. Additionally, three anatomical axes (two on the femur and one on the tibia) were assessed, as presented in Table 2.

**Figure 3:**
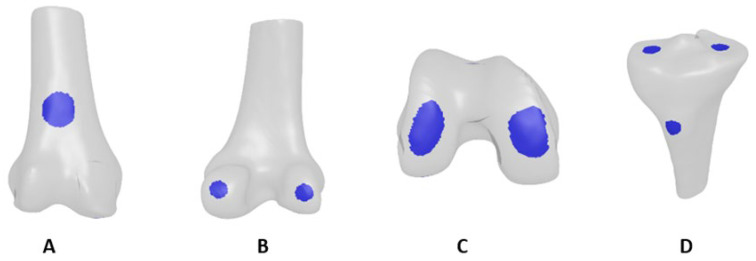
Bony landmark regions. (**A**) Femur anterior cortex. (**B**) Femur posterior condyles. (**C**) Femur distal condyles. (**D**) Tibial tuberosity, medial and lateral plateaus.

To define the locations of the bony landmarks and the anatomical axes, 3D models of the X-ray-based reconstructions and the CT segmentations were annotated by three certified orthopedic surgeons using MITK software. (Version 2023.04, German Cancer Research Center, Heidelberg, Germany.) Each surgeon was presented twice with each 3D model, in a randomized order, and was blinded to the model’s origins—whether CT-based or X-ray-based. Surgeons performed annotations independently and were unaware of the study’s purpose. For each of the two presentations (of each bone model), the surgeon annotated a single point, yielding 6 points (3 surgeons × 2 presentations) per landmark per bone model. Bony landmark regions were then defined by fitting a circle with a predefined radius to all 3 × 2 = 6 point annotations of each landmark and discarding outliers. Examples of the resulting landmark regions are shown in Figure 3. The X-ray-based bony landmark regions were then compared to the CT-based models using the RMSE.

The TEA was annotated 6 times for each bone model, by asking each of the 3 surgeons to annotate the epicondyles twice (in random order, blinded), yielding 6 annotations per bone model of a 3D TEA axis, defined simply as the line connecting the epicondyles. The 3D PCA was similarly defined as the line connecting the posterior lateral condyle to the posterior medial condyle, yielding 6 annotations per bone model. The 3D MLTA was defined as the line connecting the lateral and medial tibial plateau points, yielding 6 annotations per bone model.

**Figure 4:**
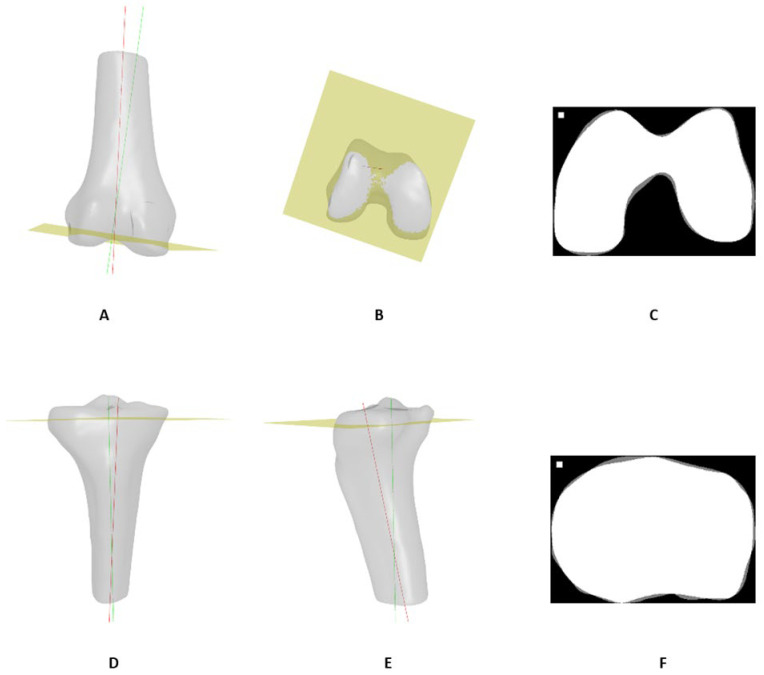
Cutting planes simulation. (**A**) Femur anatomical axis (red), estimated mechanical axis (green), and cutting plane (yellow). (**B**) Distal view of femur cutting plane. (**D**) Tibia anatomical axis (red), estimated mechanical axis (green), and cutting plane (yellow), anterior view. (**E**) Additional view of tibia cutting plane. Example cut contours of femur (**C**) and tibia (**F**) from two patients. Overlapping areas between X-ray-based and CT-based models are shown in white, and non-overlapping areas are shown in gray.

To simulate the distal femoral cutting plane, the anatomical axis of the femur was first computed by fitting a line to the center of the femoral shaft. An estimated mechanical axis was then defined at a 5° rotation angle in the AP plane. The simulated cutting plane was defined as perpendicular to the estimated mechanical axis at an offset of 9 mm from the most distal point on the medial condyle. A proximal tibial cutting plane was defined using a tibial slope at an offset of 9 mm from the most proximal tibial plateau center. The simulated cutting planes are shown in Figure 4.

A 2-dimensional slice contour was obtained by intersecting the cutting plane with the corresponding bone model to assess model performance on the cut contours. The X-ray-based model’s cut contour was then compared to the CT-based model’s cut contour using the RMSE, which was calculated by computing the square root of the mean of the square distances between each point along the contour from the closest point of the second contour. It is noteworthy that the contours were rendered at 0.1 mm resolution (Figure 4).

**Figure 5:**
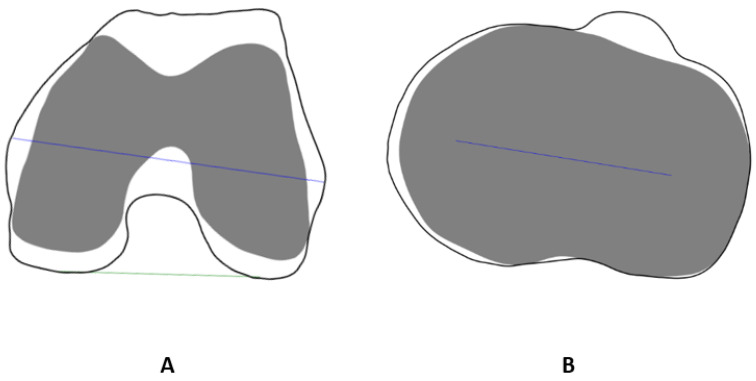
(**A**) Two-dimensional femoral axes (TEA—blue, PCA—green). The axial projection of the entire bone is shown as a black contour, and the cutting plane slice is shown in gray. (**B**) Two-dimensional MLTA with tibia projection and cut plane slice contours. TEA; trans epicondylar axis, MLTA; medial–lateral transverse axis.

The 2D anatomical axes (TEA, PCA, and MLTA) were then defined by projecting the axes that were annotated by the surgeons on the 3D models onto the relevant 2-dimensional cutting planes, as shown in Figure 5. This process yielded 6 annotations of each 2D axis per bone model.

### Statistical Analysis

RMSE values for whole bones, bony landmark regions, and cut contours were assessed by computing their means and standard deviations (STDs) across the 18 patients in the dataset.

To quantify the baseline human-level variation in anatomical axes annotated in computerized bone models, we calculated the mean angle between all possible pairs of CT-based 2D axes’ annotations for a given bone (3 × 2 = 6 annotations per bone per axis, generating 15 pairs of CT-based annotations per bone per axis), and the STD of those angles.

To assess the deviations between the 2D axes computed from X-ray-based reconstructions and the corresponding CT-based models, we first computed the angles between all possible pairs consisting of an X-ray-based axis annotation and a CT-based annotation of the corresponding axis (6 × 6 = 36 pairs per bone per axis). We then computed the means and the STDs of these 36 angles for each of the 3 axes. The statistical analysis was done using Pandas 2.1 and NumPy 1.25 Python packages.

## 3. Results

A total of 18 patients, each with one operative knee, were included in the algorithm evaluation. Patient demographics are presented in Table 3.

Global bone RMSE values for all patients are presented in Table 4. The average RMSE was 0.93 ± 0.25 mm for the femur and 0.88 ± 0.14 mm for the tibia.

**Figure 6:**
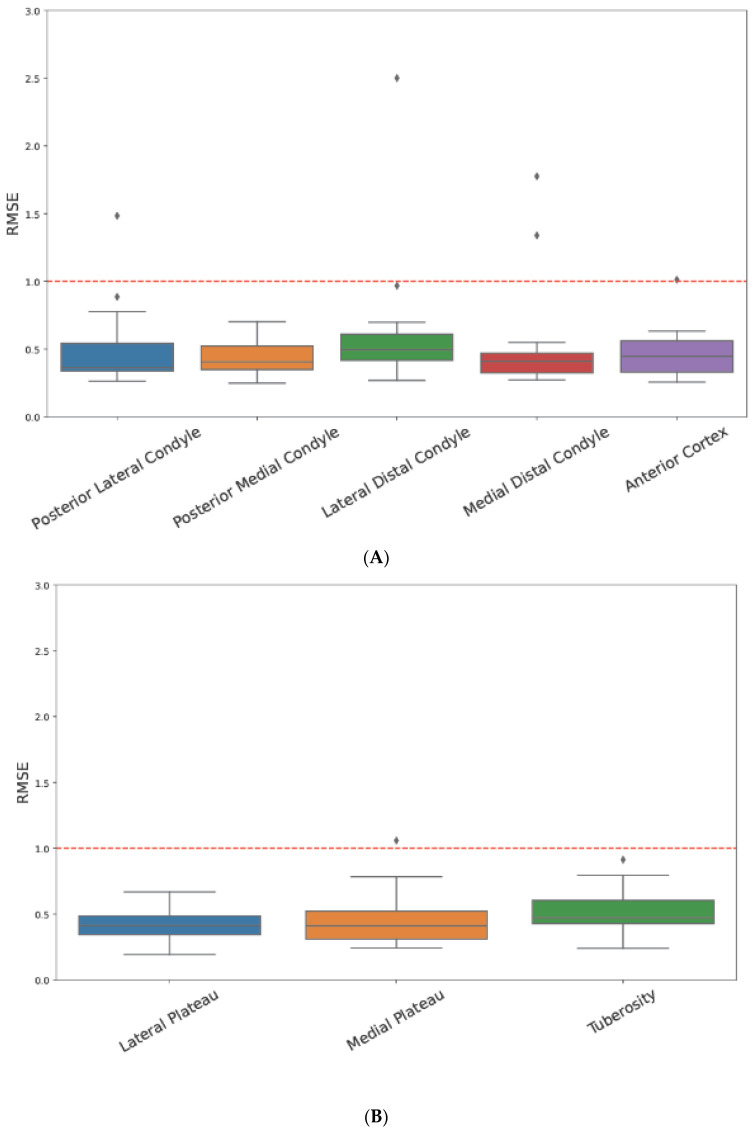
Local RMSE values of bony landmark regions. (**A**) Femoral bony landmarks. (**B**) Tibial bony landmarks. RMSE; Root Mean Square Error.

The local RMSE errors for each bony landmark region are shown in Figure 6. The mean RMSE across the five femoral landmarks in all patients was 0.51 ± 0.33 mm. The mean RMSE across the three tibial landmarks in all patients was 0.47 ± 0.17 mm.

For cut plane contours (shown in Figure 4), the mean RMSE for femoral contours across patients was 0.75 ± 0.35 mm, while the mean RMSE value for tibial contours across patients was 0.63 ± 0.27 mm.

Baseline deviation values for anatomical axes (as depicted in Figure 5) are compared to X-ray-to-CT-based deviations and presented in Table 5.

## 4. Discussion

In this study, we evaluate a novel 2D-to-3D reconstruction solution, RSIP XPlan.ai™, on data from patients that underwent TKA. Our results demonstrate the accuracy of this solution in both overall bone reconstruction as well as clinically relevant anatomical measurements typically used to plan implant size and positioning. Accuracy was consistently sub-mm, and accuracy measures on bony landmarks were in the 0.5 mm range, indicating excellent performance in clinically relevant regions. Anatomical axes were also represented in X-ray-based reconstructions with CT-level accuracy, as demonstrated by comparison to human-level baselines. 

Image-based robotics, and more generally, preoperative planning, present significant benefits to TKA workflows. Two-dimensional-to-three-dimensional reconstruction technologies present the opportunity to significantly increase access to these solutions by eliminating the necessity for a preoperative CT scan [12,16]. However, due to the paucity of information in plain-film X-rays, these technologies face significant challenges in providing the needed accuracy. In addition, these technologies are often validated on cadaveric data, which is not reflective of real-world TKA patient anatomical characteristics. For example, Fernandes et al. [9] supported further investigation into the real-world clinical value of an AI algorithm that demonstrated high accuracy in converting 2D X-rays to 3D bone models for TKA planning. When comparing the AI bone reconstructions to CT scans, four out of six anatomical measurement parameters showed mean absolute errors below 2 mm. Furthermore, when comparing the AI models to direct cadaveric bone measurements, five out of six parameters exhibited errors under 2 mm.

Victor et al. [17] evaluated the precision of determining bony landmarks on knee CT scans and concluded that these landmarks can be determined with fairly high precision. CT scans allow for reasonably precise and reproducible localization of knee anatomy, facilitating surgical planning and assessment. The mean intra-observer error was approximately 1 mm for most anatomical landmarks, indicating good repeatability. However, the mean inter-observer error ranged from 0.3 to 3.5 mm, depending on the specific landmark, suggesting moderate variability between observers. Notably, femoral epicondyles and posterior points of the tibial condyles exhibited higher inter-observer variability, with mean errors exceeding 2 mm, while joint centers and condyle centers demonstrated lower errors, typically below 1 mm.

In this work, we demonstrate that the XPlan.ai™ X-ray-based reconstructions are sub-mm accurate in clinically relevant regions when compared to CT scans. Further research is warranted to assess the accuracy of CT scans and X-ray-based models when compared to physical bones. This assessment could be conducted using tools such as a laser scanner, digitizer, or similar physical measurement apparatus.

This study has several limitations. Firstly, the data was collected exclusively from a single medical center, involving a cohort of only 18 patients. Consequently, it is advisable to conduct broader investigations to validate and establish the robustness and generalizability of the system’s performance across diverse populations and healthcare settings. It is important to note that the bone landmarks and anatomical axes were determined through surgeon interpretation rather than direct measurement from the anatomical landmarks generated from the CT scan. In addition, clinical suitability was assessed in this study by specific relevant anatomical measurements performed on computerized 3D models. Further studies are recommended for direct assessment of solution performance in the prediction of actual implant sizes and locations in the context of a real-world surgical planning workflow. Additional application-specific measures, such as registration accuracy in robotic surgery workflows, should also be explored.

## 5. Conclusions

The study findings demonstrate promising results regarding the accuracy of XPlan.ai™ in reconstructing 3D bone models from plain-film X-rays. The observed accuracy on real-world TKA patient anatomies in anatomically relevant regions, including bony landmarks, cut contours, and axes, suggests the potential utility of this method in various clinical scenarios. Further validation studies on larger cohorts are warranted to fully assess the reliability and generalizability of our results. Nonetheless, our findings lay the groundwork for potential advancements in future robotic arthroplasty technologies, with XPlan.ai™ offering a promising alternative to conventional CT scans in certain clinical contexts.

## Figures and Tables

**Table 1:** Bony landmarks.

Bone	Landmark
Femur	Posterior Lateral Condyle
	Posterior Medial Condyle
	Lateral Distal Condyle
	Medial Distal Condyle
	Anterior Cortex
Tibia	Lateral Plateau
	Medial Plateau
	Tuberosity

**Table 2:** Anatomical axes.

Bone	Axis
Femur	Trans epicondylar axis (TEA)
	Posterior condylar axis (PCA)
Tibia	Medial–lateral transverse axis (MLTA)

**Table 3:** Patient demographics.

Age (years)	71 (±8.8)
Gender, Male (n, %)	9 (50)
Height (m)	1.67 (±0.41)
Weight (kg)	86.9(±33.2)
BMI (kg/m^2^)	30.7 (±10.7)
Side—affected knee	11/7 (R/L)

**Table 4:** Global RMSE values [mm] between the X-ray-based 3D reconstructions and CT-based ground-truth 3D models for all patients’ femurs and tibias. RMSE; Root Mean Square Error, SD; standard deviation.

Patient No.	Femur	Tibia
1	0.57	0.74
2	0.97	0.94
3	0.82	0.81
4	0.77	1.03
5	0.90	0.97
6	1.04	0.79
7	0.72	0.70
8	1.21	0.98
9	1.03	1.09
10	1.57	1.09
11	1.41	0.90
12	0.75	0.84
13	0.85	0.93
14	0.87	0.88
15	0.97	0.99
16	0.72	0.86
17	0.74	0.69
18	0.87	0.62
Mean ± SD	0.93 ± 0.25	0.88 ± 0.14

**Table 5:** Baseline deviation values for anatomical axes.

	Human-Level Baseline (between-Measurement Angular Deviations, CT vs. CT)	CT vs. X-Ray Angular Deviation (between-Measurement Angular Deviations, CT vs. X-ray)
TEA	1.43° (±1.16°)	1.89° (±1.52°)
PCA	1.71° (±1.48°)	1.78° (±1.49°)
MLTA	2.56° (±1.82°)	2.82° (±2.18°)

## Data Availability

The raw data supporting the conclusions of this article will be made available by the authors on request.
